# Alemtuzumab-Induced Thyroid Dysfunction Exhibits Distinctive Clinical and Immunological Features

**DOI:** 10.1210/jc.2018-00359

**Published:** 2018-06-06

**Authors:** Nadia Pariani, Mark Willis, Ilaria Muller, Sarah Healy, Taha Nasser, Anne McGowan, Greta Lyons, Joanne Jones, Krishna Chatterjee, Colin Dayan, Neil Robertson, Alasdair Coles, Carla Moran

**Affiliations:** 1 University of Cambridge Metabolic Research Laboratories, Wellcome Trust-MRC Institute of Metabolic Science, Addenbrooke’s Hospital, Cambridge, United Kingdom; 2 Division of Psychological Medicine and Clinical Neuroscience, Department of Neurology, Cardiff University, University Hospital of Wales, Cardiff, United Kingdom; 3 Thyroid Research Group, Cardiff University, University Hospital of Wales, Cardiff, United Kingdom; 4 Department of Clinical Neurosciences, Addenbrooke’s Hospital, Cambridge, United Kingdom

## Abstract

**Context:**

Alemtuzumab, a highly effective treatment for multiple sclerosis (MS), predisposes to Graves disease (GD), with a reportedly indolent course.

**Objective:**

To determine the type, frequency, and course of thyroid dysfunction (TD) in a cohort of alemtuzumab-treated patients with MS in the United Kingdom.

**Design:**

Case records of alemtuzumab-treated patients who developed TD were reviewed.

**Results:**

A total of 41.1% (102 out of 248; 80 female and 22 male) of patients developed TD, principally GD (71.6%). Median onset was 17 months (range 2 to 107) following the last dose, with the majority (89%) within 3 years. Follow-up data (range 6 to 251 months) were available in 71 case subjects, of whom 52 (73.2%) developed GD: 10 of these (19.2%) had fluctuating TD. All 52 patients with GD commenced antithyroid drugs (ATDs): 3 required radioiodine (RAI) due to ATD side effects, and drug therapy is ongoing in 2; of those who completed a course, 16 are in remission, 1 developed spontaneous hypothyroidism, and 30 (64%) required definitive or long-term treatment (RAI, n = 17; thyroidectomy, n = 5; and long-term ATDs, n = 8). Three cases of thyroiditis and 16 cases of hypothyroidism were documented: 5 with antithyroid peroxidase antibody positivity only, 10 with positive TSH receptor antibody (TRAb), and 1 of uncertain etiology. Bioassay confirmed both stimulating and blocking TRAb in a subset of fluctuating GD cases.

**Conclusions:**

Contrary to published literature, we recorded frequent occurrence of GD that required definitive or prolonged ATD treatment. Furthermore, fluctuating thyroid status in GD and unexpectedly high frequency of TRAb-positive hypothyroidism suggested changing activity of TRAb in this clinical context; we have documented the existence of both blocking and stimulating TRAb in these patients.

## Teaser

In a retrospective analysis of alemtuzumab-induced thyroid dysfunction, Graves disease with fluctuating status that was relatively refractory to treatment and anti-TSH receptor antibody–positive hypothyroidism was recorded.

Alemtuzumab, a monoclonal antibody that binds CD52, a membrane glycoprotein on T and B lymphocytes and monocytes, leads to lysis and depletion of CD52^+^ cells (1). Its therapeutic effect is mediated by the alteration in immune repertoire that accompanies subsequent lymphocyte reconstitution (2). Alemtuzumab decreases relapse rate and disability progression in relapsing-remitting multiple sclerosis (MS), either in treatment-naive patients (3) or in patients previously treated with interferon-*β* (IFN-*β*) or glatiramer (4). Given its proven efficacy, alemtuzumab has been licensed for the treatment of relapsing-remitting MS in many regions, including the United States and European Union. It is administered intravenously, with treatment usually consisting of two courses: 12 mg/d for 5 consecutive days, followed by the same dose for 3 consecutive days 12 months later. Additional treatment courses may be considered.

The principal adverse effect of alemtuzumab is development of autoimmunity, occurring most frequently at 16 months following last date of drug administration (5). Thyroid autoimmunity is most common, with most studies reporting its occurrence in 17% to 34% of patients [41% in one study (5)]. Graves disease (GD), occurring in 60% to 70% of cases, comprises the most common cause of thyroid dysfunction (TD) (5–7). It has been suggested that individual risk is modified by smoking (threefold greater risk) and family history [sevenfold greater risk (8)]; the role of sex is uncertain, with studies suggesting no difference (8) or doubling of risk in females (9). Total alemtuzumab dosage and frequency of intervals between treatments do not appear to influence development of autoimmunity (8). The mechanism of alemtuzumab-induced autoimmunity is not fully understood, but has been attributed to a breakdown in self-tolerance during immune reconstitution postalemtuzumab, with homeostatically expanding autoreactive T cells driving a humoral autoimmune response (10). Autoimmunity is also a recognized phenomenon following immune reconstitution in other contexts, including bone marrow transplantation (11) or HIV antiretroviral therapy (12, 13); moreover, GD is the commonest form of TD seen during recovery from lymphopenia (14).

The course of alemtuzumab-induced thyroid disease is not well described, but reports suggest that GD occurring in this context may be less aggressive than the conventional disorder. In one case series (n = 31 GD), definitive treatment following failed response to antithyroid drug (ATD) therapy was only required in 26%, compared with ∼50% in conventional GD (8). Detailed analysis of TD in a large, phase 2 clinical trial showed that 23% of alemtuzumab-induced patients with GD became euthyroid spontaneously, and 15% developed hypothyroidism, with only 36% requiring radioiodine (RAI) or surgery (7, 9). In a subsequent phase 3 trial, only 2 out of 28 hyperthyroid patients required RAI or surgery (15); in another observational study, 17 of the 22 patients with GD responded to drug therapy, with only 3 cases requiring RAI (5). In contrast, anecdotal case reports suggest a poor response to ATD therapy (16), and GD with a fluctuating and unpredictable course has been noted (9).

In this study, in one of the largest case series of alemtuzumab-induced TD, followed for over 20 years, we have documented the type, frequency, and course of alemtuzumab-induced TD and determined whether response to treatment differs compared with that reported in conventional thyroid disease.

## Subjects and Methods

We undertook detailed analysis of all patients with MS who developed TD after treatment with alemtuzumab in clinical trials prior to its licensing at Addenbrooke’s Hospital in Cambridge and University Hospital Wales in Cardiff over a 20-year period (1993 to 2013).

Alemtuzumab was administered intravenously on consecutive days for one or more cycles (5 consecutive days for the first cycle and 3 consecutive days for subsequent cycles). The initial dose (20 mg/d) was increased to 24 mg/d in 2003 following a change in supplier. From 2006, the dose was reduced to 12 mg/d to conform with the phase 3 study protocol. All patients receiving alemtuzumab at either center had baseline thyroid function tests [TSH and free T4 (FT4)] prior to commencement of the drug, with TSH, FT4, and antithyroid peroxidase (TPO) antibody measurement every 3 months for 2 years, every 6 months for 2 years, and then annually, or sooner if symptoms of TD developed. At each time point, patients also underwent clinical review including directed inquiry for thyroid-related symptoms. One patient with preexisting thyroid disease, noted from her past medical history, was excluded from this analysis.

All patients who developed TD (defined later) underwent evaluation by an endocrinologist. We have reviewed clinical features at presentation and all thyroid function tests and autoantibody data and management, including response to treatment. The bioactivity of anti-TSH receptor antibody (TRAb) was measured in a subset of patients with fluctuating GD (as defined later).

### Laboratory measurements

Serum FT4, free T3 (FT3), and TSH were measured using automated immunoassay systems [ADVIA Centaur (Siemens) in Cambridge throughout and in Cardiff until 2010, with Abbott ARCHITECT thereafter].

In Cambridge, TRAb was measured initially using a first-generation ELISA assay, then LUMItest TRAK assay [reference range (RR) 1 to 2 IU/L equivocal and >2 positive; Brahms] from 2002. Cardiff also used the LUMItest TRAK assay (1 to 1.5 IU/L borderline and >1.5 IU/L positive; Brahms), changing to the Cobas assay (RR 0.9 to 1.6 IU/L borderline and >1.6 IU/L positive; Roche) in 2014. Note that, as the upper, accurately quantifiable, limit of these assays is 40 IU/L (with levels greater than this being reported as >40 IU/L), in calculations for this study, we have used 40 whenever TRAb levels of >40 were reported. Information from Thermo Fisher Scientific confirms that human TSH does not interfere with TRAb measurement in the LUMItest TRAK assay, up to TSH values of at least 500 mU/L.

Anti-TPO antibody was measured using various assays over the study period: in Cambridge, Serodia agglutination assay (positive or negative; Fujirebio Europe) up to 2002, then Phadia ELISA (RR <100 IU/mL; Phadia) until 2007, Phadia ImmunoCAP (RR <100 IU/mL; Phadia) until 2014, and then ADVIA Centaur (RR <60 IU/mL) until the present; in Cardiff, the ADVIA Centaur assay (RR <60 kU/L) until 2010 and then the Abbott ARCHITECT (RR <6 U/mL) up to the present.

The bioactivity of TRAb was measured using a Chinese hamster ovary cell line stably transfected with the human TSH receptor and a cAMP-responsive luciferase reporter, classifying them into TSH receptor-stimulating (TSAb) or TSH receptor-blocking antibody (TBAb) activities, as described previously (17, 18).

### Definitions of TD

- TD: abnormal TSH on two or more occasions, at least 3 months apart.
- GD: hyperthyroidism (low TSH with or without elevated FT4) with positive TRAb and/or increased tracer uptake (>1.5%) on technetium scan.
- Hashimoto thyroiditis (HT): raised TSH with positive anti-TPO antibody and negative TRAb.
- Thyroiditis: thyrotoxicosis followed by spontaneous euthyroidism or hypothyroidism, with negative TRAb and/or reduced or absent tracer uptake on technetium scan.
- Fluctuating GD: GD with unexpected fluctuations from hyperthyroidism to hypothyroidism (or *vice versa*), which could not be explained by omission or changes in therapy.
- TRAb-positive hypothyroidism: raised TSH with positive TRAb (with or without positive anti-TPO antibody).

## Results

**Figure 1.**
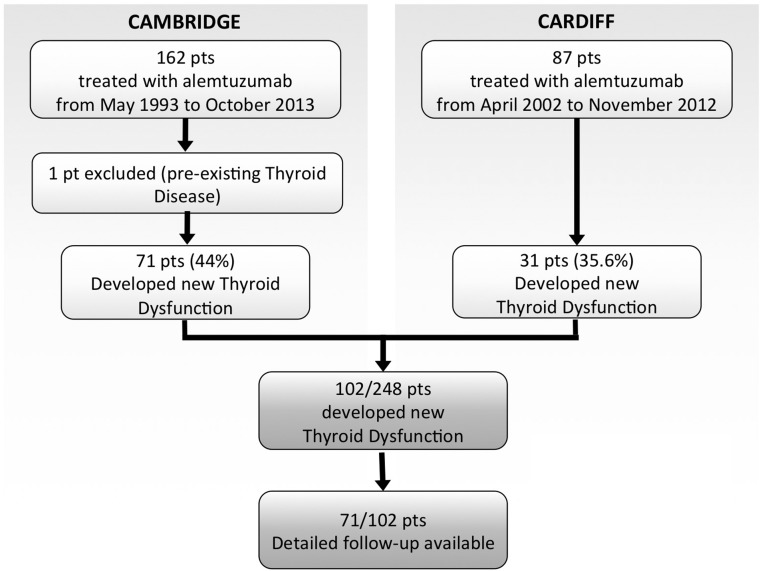
Overview of patients (pts) included in the study.

From May 1993 to October 2013, 249 patients received at least one course of alemtuzumab therapy for MS in Cambridge and Cardiff. Following this, new TD was diagnosed in 102 out of 248 (41.1%) of patients. Detailed follow-up data (mean 67 months, range 6 to 251 months) were available in 71 of these cases (Fig. 1).

### Patient characteristics

The age range of patients (n = 102) was between 20 and 60 years (mean 37.6 years), with a preponderance of females [female, n = 80 (78%); male, n = 22 (22%); female to male ratio 3.6:1] (Table 1). Most patients received more than one course of alemtuzumab treatment [courses (number of patients): 1 (n = 10); 2 (n = 55); 3 (n = 25); 4 (n = 10); and 5 (n = 2)]. A total of 34 (46.6%) patients had not received other therapy prior to alemtuzumab, and 39 (53.4%) had received other therapies (usually steroids, IFN-*β*, or glatiramer), with no prior treatment information in 29 cases. With the exception of a single case in which IFN-*β* was commenced 4 months before, none of the patients had received immunomodulatory therapy within 1 year prior to onset of TD. Anti-TPO antibody levels were checked prior to alemtuzumab in 50 patients, being negative in 42 and positive (mostly weakly) in 8 cases; TRAb levels were not tested prior to alemtuzumab.

**Table 1.** Patient Demographics and Nature of TD

	All Patients	Cases With Follow-up Data
Number	102	71
Sex		
Male, n (%)	22 (22)	18 (25)
Female, n (%)	80 (78)	53 (75)
Age, y, mean ± SD (range)	37.6 ± 9.2 (20–60)	37.8 ± 9.8 (20–60)
Number of treatment courses of alemtuzumab		
1	10	6
2	55	38
3	25	19
4	10	7
5	2	1
Interval to onset of TD after previous dose of alemtuzumab, mo, mean ± SD (range)	22.9 ± 18.2 (2–107)	23.1 ± 20.2 (2–107)
Type of TD, n (%)		
GD	73 (71.6)	52 (73.2)
Hypothyroidism with positive TRAb	12 (11.7)	10 (14.1)
HT	6 (5.8)	5 (7.0)
Thyroiditis	5 (4.9)	3 (4.2)
Hypothyroidism, unspecified	2 (2)	1 (1.4)
Hyperthyroidism, unspecified	2 (2)	
Unknown	2 (2)	

### Characteristics of TD

Overall, 41.1% (102 out of 248; 80 female and 22 male) of patients developed TD. The onset of TD, calculated in months from the date of alemtuzumab dose immediately prior to the onset of TD, was very variable (mean onset ± SD, 23 ± 18.2 months; range 2 to 107 months), with the majority of patients (89 out of 100, 89%) developing TD within 3 years of last treatment (in 2 patients, the timing of onset was unknown).

A total of 73 patients (71.6%) developed GD, 12 patients (11.7%) exhibited hypothyroidism with positive TRAb, HT occurred in 6 patients (5.8%), thyroiditis in 5 patients (4.9%), and hypothyroidism (TRAb negative, anti-TPO antibody negative, or not tested) and hyperthyroidism (TRAb negative or not tested; technetium scan not done) of unknown etiology each occurred in 2 patients; the cause of TD in 2 patients was unknown, and they were lost to follow-up (Table 1).

TRAb levels, ascertained in 72 of the 73 patients with GD, were recorded as either positive in 11 or quantified (mean ± SD TRAb level, 19.2 ± 14.7 IU/L) in 60 cases. In two patients with negative or unknown TRAb status, tracer uptake in isotope scans was diffusely increased.

### Fluctuating GD

Twelve of the 73 (16.4%) GD cases exhibited fluctuating thyroid status, transitioning from hypothyroidism to hyperthyroidism and *vice versa* after a variable period of time (Table 2). Measurement of TRAb bioactivity in eight of these patients showed the presence of both TSAb and TBAb circulating TRAb activities (Table 2).

**Table 2.** Subset of Patients With Fluctuating GD (n = 12)

Patient No.	Alemtuzumab Cycles *^a^*	Episodes of TD					
		Onset *^b^*	Type	TFT Results	TRAb (IU/L)	TRAb Bioactivity	Outcome
1	0/12/—/—	5 mo	Subclinical hypothyroidism	TSH **5.7** mU/L, FT4 10.9 pmol/L*^c^*	ND	TSAb +, TBAb +/−	Remission
		29 mo later	Hyperthyroidism	TSH **<0.03** mU/L, FT4 **25** pmol/L*^c^*	**3.4** *^c^*	TSAb ++, TBAb −	
2	0/20/—/—	18 mo	Hypothyroidism	TSH **19.30** mU/L, FT4 **10.3** pmol/L*^d^*	**>40** *^c^*	TSAb +/−, TBAb ++	Poor control on ATD necessitated RAI
		22 mo later	Hyperthyroidism	TSH **<0.03** mU/L, FT4 **42.8** pmol/L*^d^*	**>40** *^c^*	TSAb −, TBAb +	
3	0/12/—/—	6 mo	Hyperthyroidism	TSH **<0.03** mU/L, FT4 **20.9** pmol/L*^c^*	ND	TSAb +/−, TBAb −	Hypothyroidism (5 mo after stopping ATD)
		3 mo later	Hypothyroidism	NA	ND	TSAb ++, TBAb ++	
		18 mo later	Hyperthyroidism	TSH **<0.03** mU/L, FT4 **28** pmol/L*^d^*	**5.5** *^c^*	TSAb +, TBAb −	
		19 mo later	Hypothyroidism	TSH **27.1** mU/L, FT4 **10.2** pmol/L*^c^*	ND	ND	
4	0/14/82/—	12 mo	Hyperthyroidism	TSH **<0.03** mU/L, FT4 **28** pmol/L*^c^*	ND	TSAb −, TBAb −	Remission
		3 mo later	Hypothyroidism	TSH **13.40** mU/L, FT4 **10.7** pmol/L*^d^*	ND	TSAb −, TBAb +/−	
		3 mo later	Hyperthyroidism	NA	NA (**7.0**,*^c^* 5 mo later)	TSAb ++, TBAb +/−	
5	0/12/—/—	12 mo	Hypothyroidism	TSH **30.10** mU/L, FT4 **10.5** pmol/L*^d^*	ND	TSAb ++, TBAb ++	Relapse (4 mo after stopping ATD)
		48 mo later	Hyperthyroidism	TSH **<0.03** pmol/L, FT4 **41.6** pmol/L*^c^*	ND (**8.1**,*^c^* 7mo earlier)	TSAb −, TBAb +/−	
6	0/12/53/99	25 mo	Subclinical hyperthyroidism	TSH **<0.03** mU/L, FT4 19.5 pmol/L*^c^*	ND	TSAb +/−, TBAb +	Poor control on ATD
		3 mo later	Hypothyroidism	TSH **18** mU/L, FT4 **10** pmol/L*^c^*	ND	TSAb +, TBAb ++	
		20 mo later	Hyperthyroidism	TSH **<0.03** mU/L, FT4 **31.9** pmol/L*^c^*	**8.1** *^c^*	TSAb −, TBAb +/−	
7	0/12/—/—	12 mo	Hypothyroidism	TSH **98** mU/L, FT4 **5.2** pmol/L*^e^*	**>40** *^e^*	ND	Poor control on ATD necessitated RAI
		12 mo later	Hyperthyroidism	TSH **<0.01** mU/L, FT4 **32.6** pmol/L*^e^*	ND	ND	
8	0/12/39/—	11 mo	Subclinical hyperthyroidism	TSH **0.02** mU/l, FT4 14.9 pmol/L, FT3 **7.0** pmol/L*^e^*	ND	ND	Poor control on ATD necessitated RAI
		17 mo	Hypothyroidism	TSH **72** mU/L, FT4 **5.8** pmol/L*^e^*	**>40** *^e^*	ND	
		10 mo later	Hyperthyroidism	TSH **0.01** mU/L, FT4 **19.7** pmol/L, FT3 **8.3** pmol/L*^e^*	ND	ND	
9	0/12/—/—	20 mo	Hyperthyroidism	TSH **<0.01** mU/L, FT4 **26** pmol/L*^e^*	ND	ND	Poor control on ATD (awaiting second RAI treatment)
		4 mo later	Hypothyroidism	TSH **69.2** mU/L, FT4 **5.2** pmol/L*^e^*	**>40** *^e^*	ND	
		18 mo later	Hyperthyroidism	TSH **0.01** mU/L, FT4 **46** pmol/L, FT3 **24.3** pmol/L*^e^*	ND	ND	
10	0/12/—/—	9 mo	Hypothyroidism	TSH **>100** mU/L, FT4 **5.2** pmol/L*^d^*	ND	TSAb ++, TBAb ++	Remission
		18 mo later	Hyperthyroidism	TSH **<0.03** mU/L, FT4 **36.4** pmol/L*^c^*	**>40** *^c^* (date unknown)	TSAb ++, TBAb +	
11	0/12/—/—	37 mo	Hypothyroidism	TSH **6.9** mU/L, FT4 **9.7** pmol/L*^c^*	ND	TSAb +/−, TBAb −	Relapse (7 mo after stopping ATD)
		5 mo later	Hyperthyroidism	TSH **<0.03** mU/L, FT4 **44.9** pmol/L*^f^*	**Positive** *^f^*	ND	
12	0/12/—/—	12 mo	Subclinical hyperthyroidism	TSH **0.18** mU/L, FT4 **18.9** pmol/L, FT3 6 pmol/L*^c^*	ND	ND	Continuing trial of medical therapy (month 9 of a titration regimen)
		3 mo later	Hypothyroidism	TSH **23** mU/L, FT4 10.4 pmol/L*^c^*	>40*^c^*	ND	
		16 mo later	Hyperthyroidism	TSH **<0.03** mU/L, FT4 **28.6** pmol/L*^c^*	>40*^c^*	ND	

TRAb bioactivity results: −, negative; +/−, borderline; +, positive (low signal); and ++, positive (high signal).

Abbreviations: TFT, thyroid function test; NA, not available; ND, not done.

^a^ Months of administration of alemtuzumab, with 0 denoting the first cycle and subsequent cycles timed in months from administration of first cycle.

^b^ Months from the previous dose of alemtuzumab at time of initial finding of thyroid dysfunction.

^c^ RR: TSH 0.35–5.5 mU/L, FT4 10–19.8 pmol/L, FT3 3.5–6.5 pmol/L, and TRAb >1 IU/L positive. Results in boldface are outside the RR.

^d^ RR: TSH 0.35–5.5 mU/L, FT4 11.5–22.7 pmol/L. Results in boldface are outside the RR.

^e^ RR: TSH 0.35–5.0 mU/L, FT4 9–19 pmol/L, FT3 2.6–5.7 pmol/L, and TRAb >1.5 mU/L positive. Results in boldface are outside the RR.

^f^ RR unknown.

**Figure 2.**
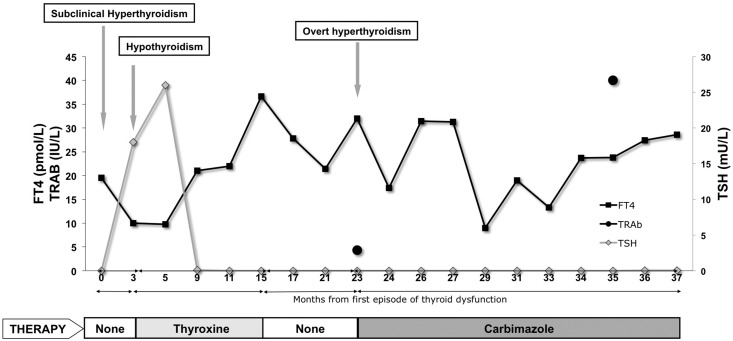
Course of TD in a patient (patient 6 in Table 3) with fluctuating GD.

The course of fluctuating thyroid status in one such case (patient 6, Table 2) is detailed in Fig. 2. Following three cycles of alemtuzumab treatment (2006, 2007, and 2011), a 44-year-old female developed subclinical hyperthyroidism (TSH <0.03 mU/L and FT4 19.5 pmol/L) in 2013, 25 months after her last treatment; she then became hypothyroid (TSH 18 mU/L and FT4 10 pmol/L) spontaneously 3 months later. Following T4 replacement for 1 year, she developed hyperthyroidism (TSH <0.03 mU/L and FT4 36.6 pmol/L), which persisted (TSH <0.03 mU/L and FT4 32 pmol/L) despite T4 withdrawal and was associated with elevated TRAb levels (initially 4.3 mU/L and then >40 mU/L): she then commenced carbimazole. Despite compliance with a block and replace (carbimazole 40 mg and thyroxine 25 μg) regimen, she remained persistently thyrotoxic (TSH <0.03 mU/L and FT4 28.6 pmol/L) and has opted to continue on high-dose thionamide (carbimazole 30 mg) therapy rather than have definitive treatment.

### TRAb-positive hypothyroidism

Twelve patients (11.7%) developed hypothyroidism associated with surprisingly high (mean 30.4 IU/L, range 3.9 to >40 IU/L) TRAb levels and variable anti-TPO antibody status (positive, n = 6, negative, n = 4; and unknown, n = 2). Measurement of TRAb bioactivity showed circulating TBAb in three out of four such cases (Table 3).

**Table 3.** Antibody Profiling in Patients With Hypothyroidism and Positive TRAb

Patient No.	Alemtuzumab Cycles *^a^*	Onset of Hypothyroidism *^b^*	TFT Results *^c^*	TPO Antibody RR 0–100 IU/mL	TRAb RR 0–1 IU/L	TSH Receptor Antibody Bioactivity
1	0/11/26/—	Month 14	TSH **34** mU/L, FT4 **8.7** pmol/L	Negative	**9.4**	TSAb +/−, TBAb +
2	0/12/123/—	Month 31	TSH **50.5** mU/L, FT4 **9.4** pmol/L	**1498**	**>40**	TSAb ++, TBAb +
3	0/12/—/—	Month 20	TSH **>100** mU/L, FT4 **5** pmol/L, FT3 **3** pmol/L	ND	**11.4**	TSAb +, TBAb ++
4	0/12/—/—	Month 11	TSH **17.6** mU/L, FT4 **4.0** pmol/L, FT3 **1.52** pmol/L	43	**>40**	TSAb +, TBAb −

TSH receptor antibody bioactivity results: −, negative; +/−, borderline; +, positive (low signal); ++, positive (high signal).

Abbreviations: ND, not done; TFT, thyroid function test.

^a^ Months of administration of alemtuzumab, with 0 denoting the first cycle and subsequent cycles timed in months from administration of first cycle.

^b^ Months from the last dose of alemtuzumab.

^c^ RR: TSH 0.35–5.5 mU/L, FT4 10–19.8 pmol/L, FT3 3.5–6.5 pmol/L. Results in boldface are outside the RR.

### Outcome of TD

To determine the course of TD, we analyzed a dataset from 71 patients in whom detailed information from follow-up (median follow-up 67 months, range 6 to 251 months) was available. The demographics of this subset of cases were similar to those of the whole TD cohort (Table 1), as were the type and frequency of TD; the majority (n = 52; 73.2%) of patients developed GD, 10 patients (14.1%) exhibited hypothyroidism with positive TRAb, HT occurred in 5 patients (7.0%), thyroiditis in 3 patients (4.2%), and hypothyroidism of unknown etiology in 1 patient. Seven (13.4%) patients (three smokers and four nonsmokers) developed clinically overt ophthalmopathy, which was particularly severe in two cases, both nonsmokers (possibly linked to RAI treatment without steroid cover in one individual), requiring immunosuppressive treatment or surgical decompression. Pretibial myxedema or acropachy was not recorded in any cases.

**Figure 3.**
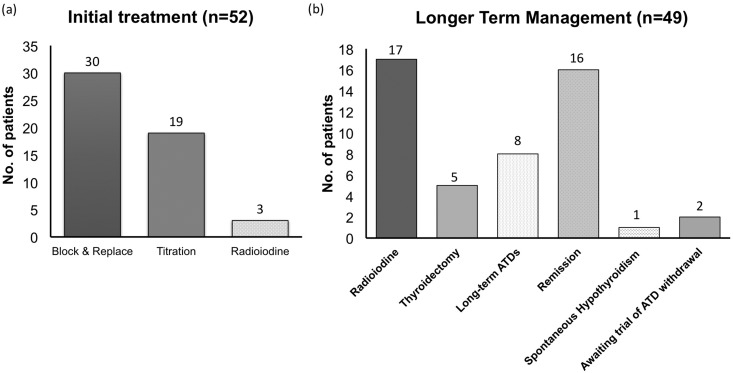
(a) Initial treatment modality in 52 patients with GD and follow-up data. Three patients underwent RAI treatment due to intolerance of ATDs. (b) Longer-term management in 47 patients with GD following completion of initial course of ATD therapy.

All 52 patients with GD were treated initially with ATD; 3 patients intolerant (rash and fatigue) of drug therapy underwent RAI within 6 months of diagnosis; 49 patients were treated with either block and replace (n = 30) or titration (n = 19) regimens [Fig. 3(a)], with the first course of ATD therapy ongoing in 2 patients [Fig. 3(b)] at time of data analysis.

Of 47 patients completing ATD treatment of appropriate duration (block and replace regimen at least 6 months; titration regimen at least 12 months), 30 (64%) individuals ultimately required definitive treatment [Fig. 3(b)]. Of these, 17 had RAI (one treatment, n = 14; two treatments, n = 2; and three treatments, n = 1), 5 underwent thyroidectomy, and 8 opted to remain on ATDs long-term (average duration 45 months, range 15 to 90 months). Reasons for RAI included relapsed (n = 10), fluctuating (n = 4), or difficult to control (n = 3) GD; thyroidectomy was undertaken in either difficult to control (n = 2) cases or patients requiring prolonged ATD treatment (n = 3; 3, 4, and 6 years on ATDs).

A total of 16 patients being followed after discontinuation of ATD treatment (average duration 82.6 months, range 28 to 137 months) remain in remission. One patient with fluctuating disease (patient 3 in Table 2) developed hypothyroidism spontaneously. Prior to discontinuation of ATD treatment, TRAb levels were only checked in seven patients (six TRAb negative, one relapsed; and one TRAb positive, relapsed).

## Discussion

TD occurred frequently (41%) in our cohort of alemtuzumab-treated patients with MS, with GD (72%) being the most frequent thyroid disorder, in accordance with previous studies (8, 9). Although TD was more commonly seen in women (female to male ratio 3.6:1), we acknowledge that the known excess female preponderance of MS could have influenced this sex distribution. Virtually all of our patients had not been treated with other MS therapies in the year preceding onset of TD.

The onset of TD was highly variable, but 89% occurred within 36 months of last administration of alemtuzumab and 91% within 4 years, the period recommended for regular thyroid surveillance. In a previous clinical trial, risk of autoimmune dysfunction peaked at 12 to 18 months after last alemtuzumab treatment, with no recorded autoimmunity beyond 5 years after therapy (8). In contrast, in our cohort, 9 patients exhibited late-onset TD (n = 2 at 5 years; n = 4 at 6 years; n = 1 at 7 years; and n = 2 at 9 years) following the last dose of alemtuzumab. Although such dysfunction might be unrelated to alemtuzumab treatment, it may be prudent to consider surveillance for TD (*e.g.*, annual TSH measurement) for a longer period following alemtuzumab therapy.

A considerable proportion (16.4%) of our patients developed GD with a fluctuating and unpredictable course, and it is conceivable that this is an underestimate, as frequent use of a block and replace ATD regimen may have masked additional cases. Fluctuating course in alemtuzumab-induced GD has been noted anecdotally previously, with one study documenting hypothyroidism followed by hyperthyroidism in four patients and unusual spontaneous transition of GD to euthyroidism or hypothyroidism (9). Measurements of TRAb bioactivity, documenting the presence of both TSAb and TBAb TRAb activities in our patients, support the notion that changes in the circulating proportions of TSAb and TBAb species over time, with resultant stimulation or inhibition of thyroid hormone production, lead to fluctuation in thyroid status. This phenomenon has been described previously (19) but in other contexts, with switching between TBAb and TSAb (or *vice versa*) being documented in rare patients following levothyroxine therapy for hypothyroidism or after ATD treatment of conventional GD (20). We have also recorded a higher prevalence (11.7%) of hypothyroidism with positive TRAb in alemtuzumab-treated patients than in conventional HT (5%) (21), suggesting that TBAb activity may also operate in this context.

Similar to the management of conventional GD in our centers, the majority (49 out of 52) of our patients with alemtuzumab-induced GD were treated with ATDs, but a higher proportion (64%) of patients proceeded to either definitive treatment (RAI or thyroidectomy) or opted to remain on ATDs long-term, compared with the proportion (50%) of patients with conventional GD exercising these options (22). In our retrospective analyses, reasons for long-term ATD treatment were not always documented, but it is likely that relative refractoriness to drug treatment or presence of a fluctuating course prompted many physicians to not withdraw ATDs at completion of a course of standard duration. Such requirement for either definitive or long-term ATD treatment and a lower remission rate (34%) compare unfavorably with the remission rate (50%) in conventional GD (22). Our observations differ from the published literature, which suggests that alemtuzumab-induced GD has a more favorable outcome, with a high rate of spontaneous remission and good response to medical treatment, than the conventional disorder (23). In conventional GD, higher TRAb levels at cessation of ATD therapy are known to be associated with greater risk of relapse following drug withdrawal (24). In our study, TRAb levels were only recorded in a minority of patients at cessation of ATDs; in future studies of ATD therapy in alemtuzumab-induced GD, serial TRAb measurement could determine whether lower remission rates correlate with differences in change of TRAb levels or activity following treatment.

A total of 13.4% of patients exhibited Graves orbitopathy (GO), but this could be an underestimate, as patients did not undergo routine ophthalmological assessment or MRI imaging, such that mild or subclinical dysthyroid eye disease might not have been recorded. Development of GO following alemtuzumab therapy is documented infrequently, with occurrence of 6.25% of patients in one study (9). In the published literature of >1000 alemtuzumab-treated patients, 6 cases of GO have been recorded, but this incidence (0.6%) is also likely to be an underestimate, as ophthalmopathy was not screened for routinely. Nevertheless, sight-threatening ophthalmopathy seems to be a rare complication of alemtuzumab treatment.

Our study has several limitations. Due to its retrospective nature, data including type and onset of TD in two cases and its etiology in four cases are missing. Our study was limited to two tertiary centers, such that complex or difficult cases could be overrepresented in the cohort; in addition, in the absence of a common treatment algorithm, the influence of differences in the management of GD cannot be completely discounted. Nevertheless, our work represents documentation of alemtuzumab-induced TD in a large patient cohort, including the course, management, and outcome of GD with prolonged duration of follow-up.

## Conclusion

Alemtuzumab is a highly effective therapy for relapsing-remitting MS (number needed to treat to benefit: 5; number needed to treat for a serious adverse event: 148) (25). However, the development of thyroid autoimmunity months or years after treatment is a frequent complication, requiring ongoing biochemical surveillance for at least 4 years after alemtuzumab therapy to detect and treat TD promptly. This study suggests that alemtuzumab-induced TD, and GD in particular, can present unique challenges: in this context, GD may develop several years after alemtuzumab treatment and exhibit a fluctuating course (likely related to changing repertoire of stimulating vs blocking TRAb), with a need for definitive (RAI or surgery) or long-term ATD treatment that exceeds that in conventional GD. Following recent regulatory approval of alemtuzumab for treatment of MS, endocrinologists will be required to manage this form of TD more often. Based on our experience, we suggest close monitoring of thyroid function in patients with alemtuzumab-treated MS, particularly if they develop GD, offering early definitive treatment in drug-refractory or fluctuating cases.

## Abbreviations:

ATD: antithyroid drug
FT3: free T3
FT4: free T4
GD: Graves disease
GO: Graves orbitopathy
HT: Hashimoto thyroiditis
IFN-*β*: interferon-*β*
MS: multiple sclerosis
RAI: radioiodine
RR: reference range
TBAb: TSH receptor-blocking antibody
TD: thyroid dysfunction
TPO: antithyroid peroxidase
TRAb: TSH receptor antibody
TSAb: TSH receptor-stimulating antibody

## References

[1] Greenwood J, Gorman SD, Routledge EG, Lloyd IS, Waldmann H Engineering multiple-domain forms of the therapeutic antibody CAMPATH-1H: effects on complement lysis. Ther Immunol. 1994;1(5):247–255. 7584499

[2] Hill-Cawthorne GA, Button T, Tuohy O, Jones JL, May K, Somerfield J, Green A, Giovannoni G, Compston DA, Fahey MT, Coles AJ Long term lymphocyte reconstitution after alemtuzumab treatment of multiple sclerosis. J Neurol Neurosurg Psychiatry. 2012;83(3):298–304. 22056965 10.1136/jnnp-2011-300826

[3] Havrdova E, Arnold DL, Cohen JA, Hartung HP, Fox EJ, Giovannoni G, Schippling S, Selmaj KW, Traboulsee A, Compston DAS, Margolin DH, Thangavelu K, Rodriguez CE, Jody D, Hogan RJ, Xenopoulos P, Panzara MA, Coles AJ; CARE-MS I and CAMMS03409 Investigators Alemtuzumab CARE-MS I 5-year follow-up: Durable efficacy in the absence of continuous MS therapy [published correction appears in *Neurology* 2018;**90**(16):755]. Neurology. 2017;89(11):1107–1116. 28835401 10.1212/WNL.0000000000004313 PMC5595278

[4] Coles AJ, Cohen JA, Fox EJ, Giovannoni G, Hartung HP, Havrdova E, Schippling S, Selmaj KW, Traboulsee A, Compston DAS, Margolin DH, Thangavelu K, Chirieac MC, Jody D, Xenopoulos P, Hogan RJ, Panzara MA, Arnold DL; CARE-MS II and CAMMS03409 Investigators Alemtuzumab CARE-MS II 5-year follow-up: Efficacy and safety findings. Neurology. 2017;89(11):1117–1126. 28835403 10.1212/WNL.0000000000004354 PMC5595276

[5] Tuohy O, Costelloe L, Hill-Cawthorne G, et al Alemtuzumab treatment of multiple sclerosis: long term safety and efficacy. J Neurol Neurosurg Psychiatry. 2015;86(2):208–215. 24849515 10.1136/jnnp-2014-307721

[6] Coles AJ, Wing M, Smith S, Coraddu F, Greer S, Taylor C, Weetman A, Hale G, Chatterjee VK, Waldmann H, Compston A Pulsed monoclonal antibody treatment and autoimmune thyroid disease in multiple sclerosis. Lancet. 1999;354(9191):1691–1695. 10568572 10.1016/S0140-6736(99)02429-0

[7] Coles AJ, Compston DA, Selmaj KW, Lake SL, Moran S, Margolin DH, Norris K, Tandon PK; CAMMS223 Trial Investigators Alemtuzumab vs. interferon beta-1a in early multiple sclerosis. N Engl J Med. 2008;359(17):1786–1801. 18946064 10.1056/NEJMoa0802670

[8] Cossburn M, Pace AA, Jones J, Ali R, Ingram G, Baker K, Hirst C, Zajicek J, Scolding N, Boggild M, Pickersgill T, Ben-Shlomo Y, Coles A, Robertson NP Autoimmune disease after alemtuzumab treatment for multiple sclerosis in a multicenter cohort. Neurology. 2011;77(6):573–579. 21795656 10.1212/WNL.0b013e318228bec5

[9] Daniels GH, Vladic A, Brinar V, Zavalishin I, Valente W, Oyuela P, Palmer J, Margolin DH, Hollenstein J Alemtuzumab-related thyroid dysfunction in a phase 2 trial of patients with relapsing-remitting multiple sclerosis. J Clin Endocrinol Metab. 2014;99(1):80–89. 24170099 10.1210/jc.2013-2201

[10] Jones JL, Thompson SA, Loh P, Davies PL, Tuohy OC, Curry AJ, Azzopardi L, Hill-Cawthorne G, Fahey MT, Compston A, Coles AJ Human autoimmunity after lymphocytte depletion is caused by homeostatic T-cell proliferation. Proc Natl Acad Sci USA. 2013;110(50):20200–20205. 24282306 10.1073/pnas.1313654110 PMC3864306

[11] Daikeler T, Tyndall A Autoimmunity following haematopoietic stem-cell transplantation. Best Pract Res Clin Haematol. 2007;20(2):349–360. 17448966 10.1016/j.beha.2006.09.008

[12] Chen F, Day SL, Metcalfe RA, Sethi G, Kapembwa MS, Brook MG, Churchill D, de Ruiter A, Robinson S, Lacey CJ, Weetman AP Characteristics of autoimmune thyroid disease occurring as a late complication of immune reconstitution in patients with advanced human immunodeficiency virus (HIV) disease. Medicine (Baltimore). 2005;84(2):98–106. 15758839 10.1097/01.md.0000159082.45703.90

[13] Jubault V, Penfornis A, Schillo F, Hoen B, Izembart M, Timsit J, Kazatchkine MD, Gilquin J, Viard JP Sequential occurrence of thyroid autoantibodies and Graves’ disease after immune restoration in severely immunocompromised human immunodeficiency virus-1-infected patients. J Clin Endocrinol Metab. 2000;85(11):4254–4257. 11095463 10.1210/jcem.85.11.6988

[14] Weetman A Immune reconstitution syndrome and the thyroid. Best Pract Res Clin Endocrinol Metab. 2009;23(6):693–702. 19942146 10.1016/j.beem.2009.07.003

[15] Coles AJ, Twyman CL, Arnold DL, Cohen JA, Confavreux C, Fox EJ, Hartung HP, Havrdova E, Selmaj KW, Weiner HL, Miller T, Fisher E, Sandbrink R, Lake SL, Margolin DH, Oyuela P, Panzara MA, Compston DA; CARE-MS II investigators Alemtuzumab for patients with relapsing multiple sclerosis after disease-modifying therapy: a randomised controlled phase 3 trial. Lancet. 2012;380(9856):1829–1839. 23122650 10.1016/S0140-6736(12)61768-1

[16] Tsourdi E, Gruber M, Rauner M, et al Graves’ disease after treatment with alemtuzumab for multiple sclerosis. Hormones (Athens). 2015;14:148–153. 25402383 10.14310/horm.2002.1501

[17] Evans C, Morgenthaler NG, Lee S, Llewellyn DH, Clifton-Bligh R, John R, Lazarus JH, Chatterjee VK, Ludgate M Development of a luminescent bioassay for thyroid stimulating antibodies. J Clin Endocrinol Metab. 1999;84(1):374–377. 9920111 10.1210/jcem.84.1.5532

[18] Jordan NJ, Rinderle C, Ashfield J, Morgenthaler NG, Lazarus J, Ludgate M, Evans C A luminescent bioassay for thyroid blocking antibodies. Clin Endocrinol (Oxf). 2001;54(3):355–364. 11298088 10.1046/j.1365-2265.2001.01193.x

[19] Takeda K, Takamatsu J, Kasagi K, Sakane S, Ikegami Y, Isotani H, Majima T, Majima M, Kitaoka H, Iida Y, et al Development of hyperthyroidism following primary hypothyroidism: a case report with changes in thyroid-related antibodies. Clin Endocrinol (Oxf). 1988;28(4):341–344. 2903804 10.1111/j.1365-2265.1988.tb03664.x

[20] McLachlan SM, Rapoport B Thyrotropin-blocking autoantibodies and thyroid-stimulating autoantibodies: potential mechanisms involved in the pendulum swinging from hypothyroidism to hyperthyroidism or vice versa. Thyroid. 2013;23(1):14–24. 23025526 10.1089/thy.2012.0374 PMC3539254

[21] Kahaly GJ, Diana T, Glang J, Kanitz M, Pitz S, König J Thyroid stimulating antibodies are highly prevalent in Hashimoto’s thyroiditis and associated orbitopathy. J Clin Endocrinol Metab. 2016;101(5):1998–2004. 26964732 10.1210/jc.2016-1220

[22] Abraham P, Avenell A, McGeoch SC, Clark LF, Bevan JS Antithyroid drug regimen for treating Graves’ hyperthyroidism. Cochrane Database Syst Rev. 2010; (1):CD003420. 20091544 10.1002/14651858.CD003420.pub4 PMC6599817

[23] Weetman AP Graves’ disease following immune reconstitution or immunomodulatory treatment: should we manage it any differently? Clin Endocrinol (Oxf). 2014;80(5):629–632. 24528193 10.1111/cen.12427

[24] Tun NNZ, Beckett G, Zammitt NN, Strachan MWJ, Seckl JR, Gibb FW Thyrotropin receptor antibody levels at diagnosis and after thionamide course predict Graves’ disease relapse. Thyroid. 2016;26(8):1004–1009. 27266892 10.1089/thy.2016.0017

[25] Mendes D, Alves C, Batel-Marques F Benefit-risk of therapies for relapsing-remitting multiple sclerosis: testing the number needed to treat to benefit (NNTB), number needed to treat to harm (NNTH) and the likelihood to be helped or harmed (LHH): A systematic review and meta-analysis. CNS Drugs. 2016;30(10):909–929. 27538416 10.1007/s40263-016-0377-9

